# Fluorescence Multi-Detection Device Using a Lensless Matrix Addressable microLED Array

**DOI:** 10.3390/bios14060264

**Published:** 2024-05-22

**Authors:** Victor Moro, Joan Canals, Sergio Moreno, Steffen Higgins-Wood, Oscar Alonso, Andreas Waag, J. Daniel Prades, Angel Dieguez

**Affiliations:** 1 Electronic and Biomedical Engineering Department, University of Barcelona, 08028 Barcelona, Spain; canals@ub.edu (J.C.); sergiomoreno@ub.edu (S.M.); oalonso@ub.edu (O.A.); dprades@ub.edu (J.D.P.); 2 Institute of Semiconductor Technology, Technical University of Braunschweig, 38106 Braunschweig, Germany; steffen.bornemann@tu-braunschweig.de (S.H.-W.); a.waag@tu-braunschweig.de (A.W.)

**Keywords:** Point-of-Care, multiplex, microLED array, SPAD, fluorescence, lifetime fluorescence, GaN, CMOS, microLED driver

## Abstract

A Point-of-Care system for molecular diagnosis (PoC-MD) is described, combining GaN and CMOS chips. The device is a micro-system for fluorescence measurements, capable of analyzing both intensity and lifetime. It consists of a hybrid micro-structure based on a 32 × 32 matrix addressable GaN microLED array, with square LEDs of 50 µm edge length and 100 µm pitch, with an underneath wire bonded custom chip integrating their drivers and placed face-to-face to an array of 16 × 16 single-photon avalanche diodes (SPADs) CMOS. This approach replaces instrumentation based on lasers, bulky optical components, and discrete electronics with a full hybrid micro-system, enabling measurements on 32 × 32 spots. The reported system is suitable for long lifetime (>10 ns) fluorophores with a limit of detection ~1/4 µM. Proof-of-concept measurements of streptavidin conjugate Qdot™ 605 and Amino PEG Qdot™ 705 are demonstrated, along with the device ability to detect both fluorophores in the same measurement.

## 1. Introduction

During the last decades, the increase in life expectancy has led to a global population aging, significantly increasing the demand for healthcare services for elderly individuals. According to the World Health Organization (WHO), this trend of the global population’s average age rising is anticipated to continue in the years ahead. It is expected that by 2030, 1 in 6 people worldwide will be over 60 years old (1.4 billion), and by 2050, this number is projected to reach 2.1 billion. Additionally, the number of people over 80 years old is expected to triple from 2020 to 2050. The expectation is that by 2050, 80% of older people will live in low- and middle-income countries [1]. In these environments, access to the health system is difficult due to several factors, including lack of resources, low staff pay, and lack of equipment and infrastructure, including accessibility of health services or low levels of education [2]. One outcome of inadequate access to the health system is the delay in disease diagnosis, which can be critical for saving patients’ lives and preventing the spread of infectious diseases [3,4,5,6]. Moreover, studies indicate that early detection and analysis of diseases led to a decreased time, cost, and necessity for further diagnostic procedures, for example, early detection of a disease such as influenza in children presenting with fever at emergency rooms [7].

The emergence of technologies that enhance efficiency and reduce diagnosis time has spurred the development of several rapid diagnostic platforms suitable for Point-of-Care (PoC) applications [8]. With the application of fast diagnostic methods such as PoC devices for just four common diseases–syphilis, tuberculosis, malaria, and bacterial pneumonia–it is possible to prevent 1.2 million deaths annually [9,10].

PoC takes special relevance by bringing the clinical laboratory closer to the patient and with reduced cost. This is especially relevant since the majority of the population that would require higher access to healthcare services is located in places with limited access to these services. The key features of PoC include portability, ease of use, and rapid result turnaround times. These features enable diagnosis and monitoring of diseases, and furthermore, management near to the patient, which facilitates personalized therapy and enhances patient outcomes with a reduced overall cost for the National Health Systems [11]. According to WHO, PoC tests considered appropriate for the delivery of healthcare in this resource-limited environment should meet the criteria of “ASSURED”, which stands for Affordable, Sensitive, Specific, User-friendly, Rapid and Robust, Equipment-free, and Deliverable [12,13].

A PoC device is usually formed by five components: sensing tool, transducer, target, prove, and signal readout device [14]. PoC devices have been proven to be useful in a wide range of applications, such as diagnosis of immunological [15], cardiovascular [16], infectious [12], neurodegenerative [17], and oncological diseases [18]. Moreover, they can also be used in blood [19], genetic [20], and microbiology testing [21]. All these applications are performed by different transducers, but the most common and inexpensive one is optics, specifically imaging analysis by fluorescence [22,23,24]. There are several PoC devices that use fluorescence as transduction tools in the literature. The research reports that PoC devices based on fluorescence have good efficiency and performance, and they aim to improve the limit of detection (LoD) and miniaturization. In these devices, the most used light source to produce fluorescence is a laser [25,26,27,28]. However, in recent years, the use of Light Emitting Diodes (LEDs) and microLEDs has been introduced in some PoC devices [29,30,31,32]. Additional elements of these PoC are lenses to focus light on the sample and to improve the detection of light coming from the fluorophores, emission and excitation filters, and a photodetector. 

Fluorescence-based PoCs are typically designed to identify the presence of a specific substance through intensity measurements. The intensity measurements are performed by continuously illuminating the sample that is excited by the light. If the targeted analyte is present, the sample emits fluorescence light red-shifted compared to the original excitation light. The emitted light is tracked and utilized to measure a biochemical reaction or binding occurrence, offering high accuracy, sensitivity (capable of single molecule detection), and precise labeling of biological samples [33]. Nevertheless, fluorescence techniques relying on intensity measurements are susceptible to misinterpretation because they depend on factors such as excitation light intensity and fluorophore concentration. It can be found in the literature that one of the solutions proposed to overcome these limitations is provided by time-resolved techniques, in which the lifetime or the decay of the fluorophores is measured. The lifetime of a fluorophore is an intrinsic characteristic of each molecule, and is therefore independent of the concentration or excitation intensity of the fluorophore [24,34]. In these measurements, the light source is pulsed, exciting the sample for a specified time. Once the light source is turned off, it is possible to measure the lifetime of the fluorophore. A key feature that limits the capability to detect fluorophores lifetimes is the speed at which the device can turn off the light source, limiting the minimum detectable lifetime. Moreover, the possibility of detecting fluorophore lifetimes enhances the specificity of the measurement by time domain discrimination, thus allowing us to discern the light of interest from the background noise [35]. Furthermore, it allows us to discern between different fluorophores with overlapping emission spectra but with different lifetimes in multiplexed assays [36,37,38].

In recent years, several advances have been made in PoC devices, especially using LEDs, since they are less expensive than lasers. Furthermore, the use of LEDs in arrays allows multiplexing. U. Obahiagbon et al. [39] presented a PoC using an array of 2 × 2 green LEDs to detect antibodies to HPV16 and 18 proteins. In [40], F. B. Myers et al. designed a PoC and performed an assay for the HIV integrase gene, which they were able to detect at a concentration of 10^3^ copies/µL. J. T. Smith et al. [41] used the device presented in [39] to measure a disposable 4-site fluorescent microscope slide reader with high sensitivity for LMIC disease diagnosis. Manzanas et al. [42] developed a rapid and sensitive multiplexed PoC device capable of simultaneous detection of SARS-CoV-2 and influenza A HINI viruses in 50 min with the use of a blue LED. In [43], B. Shu et al. pursued an ultraportable, automated, and multiplexed PoC molecular platform that can provide screening of infectious pathogens rapidly and with high sensitivity. The PoC device reported has the possibility to work with 15-channel performing real-time quantitative detection.

In this work, we present a PoC device that uses a GaN-based microLED array chip, instead of lasers, LEDs, or LED arrays, as an excitation source. By following the trend in LED platform development and making use of the advances in GaN-based microLED arrays, it is possible to develop a device with high multiplexity. Specially, the high brightness capabilities of the GaN-based LEDs [44,45] allow them to be a suitable substitute for the lasers that are typically used in fluorescence PoC devices. Furthermore, their high modulation bandwidth [46,47] (up to 1 GHz) makes them a perfect candidate for time-resolved fluorescence measurements. Therefore, in this work, a PoC device is built with a 32 × 32 matrix addressable (MA) microLED array and a single-photon avalanche photodiodes (SPAD) camera as the main components; that the device is able to perform both intensity and time-correlated fluorescence measurements. Moreover, this device can perform both types of measurements without any optical components.

In the subsequent sections, we describe the instrument and its components, followed by a detailed characterization of the device. This includes measurements of fluorescence intensity and fluorescent lifetimes across varying concentrations of two distinct quantum dot molecules.

## 2. Materials and Methods

### 2.1. Instrument

**Figure 1:**
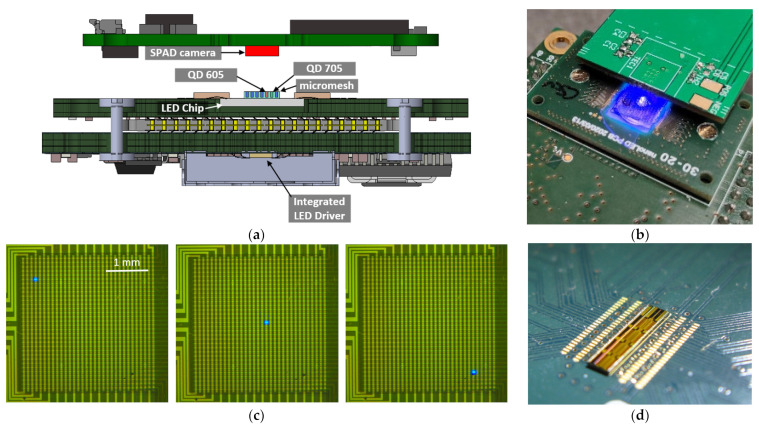
The schematic view of the setup is shown in (**a**), and a picture of the setup is in (**b**). (**c**) is a microscopic picture of the array of microLEDs with different LEDs turned on and (**d**) is a picture of the CMOS driver wire bonded to the PCB.

A device was constructed that enables the acquisition of fluorescence intensity and facilitates time-resolved experiments to assess fluorescence lifetime (Figure 1a,b). The setup built for both fluorescence methods is the same. To perform the measurements, the LED light is pulsed, and time gating is applied before the failing edge of the excitation [48,49]. For intensity measurements, the light measured after the LED is turned off is measured, thus detecting a fluorophore or background. To perform time-correlated measurements, the arrival time of the photons is measured, and the lifetime is obtained after processing the obtained histogram [50]. Thus, when performing the measurements as described, a filter is not necessary. This allows the setup to increase its miniaturization and reduce its cost, which are both key factors for PoC devices, following the “ASSURED” criteria. The main part of the setup consists of a sandwich with the microLED array (Figure 1c) on one side driven by a custom CMOS chip (Figure 1d) underneath and a custom CMOS SPAD optical sensor on top. The sample is placed in between using a micromesh. Validation of the instrument was conducted with two different quantum dots (QD605 and QD705, described in Section 2.5) with different lifetimes. The quantum dots are deposited in different wells of a micromesh plate (Section 2.6).

### 2.2. microLED Array

**Figure 2:**
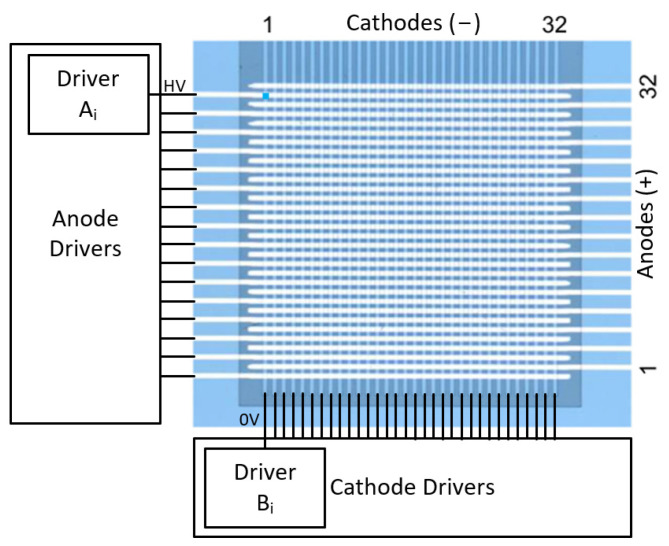
Image of the LED chip with the anodes at the right side and the cathodes at the top. As can be observed in the image, only 32 anode connections and 32 cathode connections are needed for an array of 32 × 32 LEDs [51].

The fabrication of the matrix-addressed LED arrays (Figure 1c) is described in detail in a previous publication [51]. It consists of 32 × 32 squared LEDs of 50 µm edge length edge length with 100 µm pitch (Figure 1c). This array is matrix addressable, having all the anodes in the same column connected and all the cathodes in the same row connected (Figure 2). This means the LED chip needs only 32 anode connections and 32 cathode connections to address 1024 LEDs (32 × 32). They were fabricated from standard blue LED wafers on InGaN/GaN basis, emitting at a peak wavelength of approximately 450 nm.

The microLED chip was created by etching fin structures into the GaN film. Coupled plasma reactive ion etching (ICP-RIE) was used to etch down to the sapphire substrate. A subsequent wet etch in KOH ensured smoother fin sidewalls and improved passivation. A Ti/Au-based metal pad was provided to each cathode on two opposite edges of the chip as electrical contacts. Subsequently, the trenches in between the array of fins were filled with the polymer benzocyclobuthene (BCB) that was applied by spin-coating. The following hardbake is required to cure the BCB. After this procedure, the resin covers the whole array, and careful mechanical polishing was used for removal and planarization of the BCB, until the fin surfaces were exposed again. Subsequently, an SU-8-based insulation layer was created on the planarized surface, with 32 × 32 openings that define the pixel positions on the fins. Pd/Au contact pads to the p-GaN were then deposited in the opening of the SU-8, and metal lines of Ti/Au were deposited perpendicular to the fins, each connecting a row of pixels and leading to a contact pad at the two remaining chip edges. Another SU-8 layer was then applied as encapsulation of the chip.

From matrix addressable LED chips, it is expected that the larger the number of pixels is, the larger the capacitance to drive is, since the capacitance of every LED per row (or column) is added to the anode (or cathode) node. This would affect the driving rate of the LEDs and, for a large number of pixels, a higher current will be required compared to a smaller number. However, a similar problem affects direct addressable (DA) arrays, since the resistance of the interconnection between each LED with its driver increases considerably, causing a similar RC delay [52,53].

### 2.3. Custom Driver Chip

**Figure 3:**
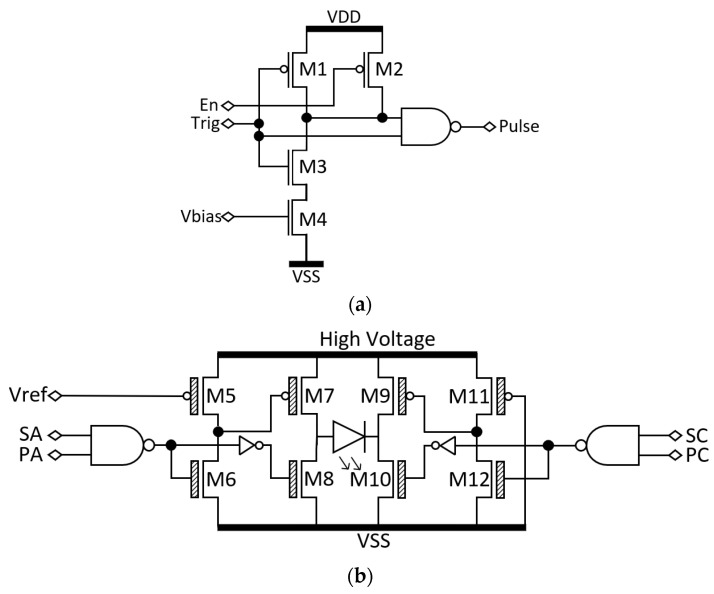
Short pulse generation circuit (**a**) and the anode and cathode driving elements (**b**). The anode and cathode driving circuits are composed of high voltage output buffers (M7–M8 for the anode driver and M9–M10 for the cathode driver) and level shifters (M5–M6 and M11–M12) with a NAND gate per driver to select the specific LED.

A chip was produced with the capability of driving the 32 × 32 matrix addressing LED array. The chip contains 32 anodes (p-contacts) and 32 cathodes (n-contacts) drivers. Each driver consists of a combination of these two main circuits (one anode driver *A_i_* and one cathode driver *B_i_*), both shown in Figure 3b. In MA, to switch on an LED, the associated row (anode) must be biased positive while the associated column (cathode) is at ground (Figure 2). The rest of the columns must be biased positive too. First, a column of cathodes *B_i_* (Figure 2) is selected by switching the voltage to 0 V, thus allowing us to drive the LEDs in that column. Then, the LEDs in each row are turned on and off by switching the anode drivers *A_i_* (Figure 2). These circuits are designed so the critical node is the anode, which determines the rate at which the LED is charged/discharged.

**Figure 4:**
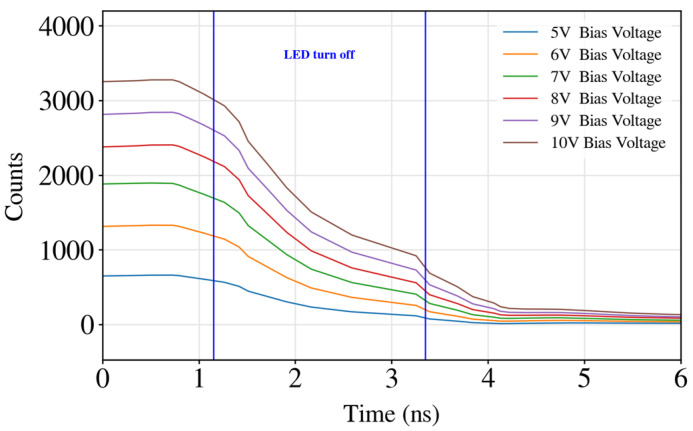
Driving circuit turning off a microLED for different bias voltages. It can be observed that the turn off time is the same for all the bias voltage, therefore making the circuit speed robust to changes in the driving voltage of the LEDs. The y axis (Counts) represents the optical intensity captured by the SPAD sensor.

The driver can operate up to 10 V, thus allowing the LED to provide high optical power (~30 µW at 6 V [51]). The capability of these drivers to generate driving voltages up to 10 V also enables the circuit to be used to drive nanoLEDs [54], which usually work at a higher voltage bias [55] because of the high resistance associated with the interconnection of the LED with the CMOS [54]. In the matrix addressing LED array of this work, the driver can turn off an LED in 2 ns (Figure 4), thus allowing this circuit to be used in time-resolved fluorescence.

Each anode driving pixel measures 572 × 95 µm^2^ and contains a low-voltage short pulse generator (Figure 3a) and the high-voltage driving circuit (Figure 3b M5–M8). The cathode driving pixel measures 175 × 115 µm^2^, has the low-voltage short pulse generator (Figure 3a), and the high-voltage driving circuit (Figure 3b M9–M12). The short-pulse generator consists of an AND gate between an external input signal (*Trig*) and its delayed and inverted version. The width of the pulse (*PA* for the anode and *PC* for the cathode) is controlled by a bias voltage (*V_bias_*) that changes the resistance of M4. To allow longer pulses, M2 is driven by an enable (*En*) signal, which disables the circuit, allowing the use of an external signal to drive the LED. The anode driver consists of a level shifter (M5 and M6) and a high voltage inverter (M7 and M8) with a high W/L ratio that allows fast charge/discharge of the LEDs. The cathode driver consists of a level shifter (M11 and M12) and a high-voltage inverter (M9 and M10). 

To measure the response time of the fluorophore and to calculate its decay time constant (or lifetime), the sample must be excited with a light source that is able to turn off as fast as possible. The faster the driver can turn off the light source, the shorter the decay times of the fluorophores the device would be able to measure. Figure 4 shows the results of performing time-correlated single photon counting with single LED pulses to observe its rapid response. To conduct such measurements, the SPAD camera described in the following section was used. As can be observed in Figure 4, the CMOS driving circuit is designed to be able to perform fast transitions, thus enabling it to harness the rapid response that GaN LEDs provides. In this case, it is proven that the LED can be turned off in the 2 ns range for all the bias voltages (calculated from 90% to 10% of the maximum signal), which allows the device to perform detections of fluorophores with lifetimes down to the range of several ns.

### 2.4. SPAD Camera

Details about the custom CMOS SPAD camera, designed in Barcelona, Spain, and manufactured by Austria MicroSystems, Premstaetten, Austria, can be found in a previous publication [56]. It was manufactured in a 0.35 µm High-Voltage (HV) CMOS process. The camera consists of 16 × 16 circular SPAD sensors with 10 µm diameter and a pitch of 70 µm. The camera has low noise, with a Dark Count Rate (DCR) below 1 kHz for 90% of the pixels at the working conditions (19 V breakdown voltage, with an overvoltage of 1.3 V). However, for the experiments performed here, one pixel is selected with a DCR of only 300 Hz. The Photon Detection Probability (PDP) of the chip is 12% centered in 570 nm. For timing measurements, a SPAD sensor provides a time resolution in the order of ps [57,58]. Acquisition in this work is performed with an external FPGA (ZedBoard Zynq.7000, purchased from Digilent, Pullman, WA, USA [59]) with a minimum bin resolution of 68 ps and a maximum number of bins of 6402 [60].

### 2.5. Fluorescent Particles

QDot^®^ 605 ITK™ Streptavidin [61] and QDot^®^ 705 ITK™ Amino PEG [62], referred to as QD605 and QD705, respectively, were both purchased from Life Technologies, Waltham, MA, USA. D705 is equipped with amine-derivatized polyethylene glycol (PEG) ligands covalently bonded to an amphiphilic coating, which enhances their water solubility and facilitates the conjugation of biomolecules. This conjugation is enabled through the reactive amino groups via N-hydroxysuccinimide (NHS) esters. QD605 is comprised of a biotin-binding protein linked to a fluorescent label. Due to its high affinity for biotin, streptavidin in QD605 is typically used with biotinylated conjugates for the targeted detection of various proteins, protein motifs, nucleic acids, and other biomolecules.

The maximum emission peaks for QD605 and QD705 are in 605 nm and 705 nm, respectively. Both are excited in the UV but have a reasonable excitation, with maximum emission at 450 nm for the LEDs used in this work around 20%. QD605 has an expected lifetime in the order of 32 ns, reported by J. Canals et al. [24]. QD705 has an expected lifetime of around 80 ns, as reported in [63] by S. Bhuckory et al.

### 2.6. Micromesh

To distribute the fluorophores in known distance zones, a micromesh was used. The micromesh was purchased from Tebu-bio Spain S.L., Barcelona, Spain [64]. Microwell diameter is 250 µm with 500 µm pitch. In the experiments, a volume of 5 nL QD was loaded in selected microwells of the micromesh.

## 3. Results

### 3.1. Intensity of Fluorescence Measurements

**Figure 5:**
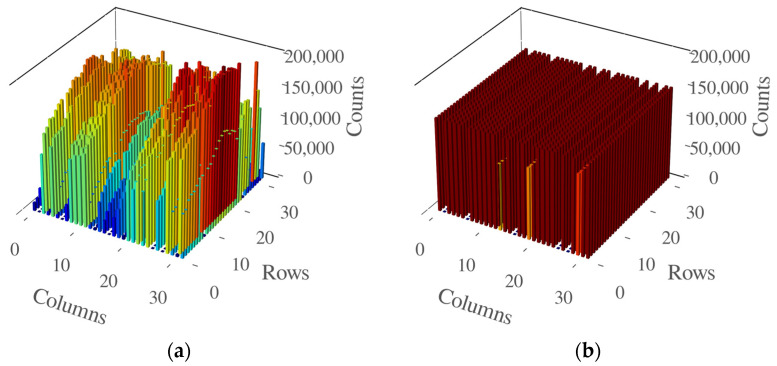
LEDs at 6V bias voltage (**a**) without any calibration. Each LED emits different power. In (**b**) all the LEDs were calibrated to 150 kcounts. In (**b**) there are visible non-working LEDs.

For fluorescence intensity measurements, the LEDs were initially calibrated to provide the same optical output, measured as 150,000 counts in the SPAD. The SPAD sensor measured 255,000 times, with windows of detection of 200 ns for each LED. In Figure 5, we present the map of the intensity the LEDs have at 6 V bias voltage (Figure 5a) and the equalization for 150,000 counts (Figure 5b).

**Figure 6:**
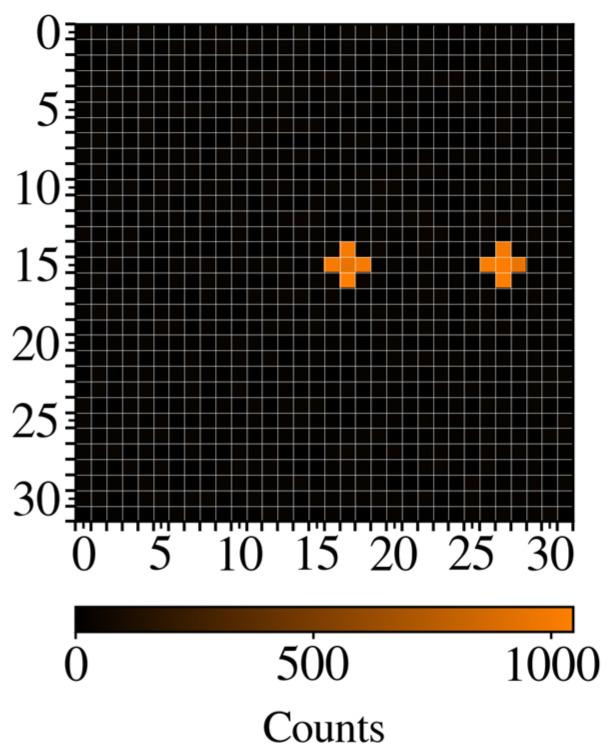
Image acquired by the device, where QD605 is detected in the orange areas (above 1000 counts in each one). The other part of the image corresponds to absence of QD605.

With the LEDs calibrated, the experiment was performed. A micromesh with QD605 placed in two microwells was positioned on top of the microLED array. The measurement for each LED was performed for 2 ms. In this period, the LED was pulsed 10,000 times. First, the LED was switched on for 130 ns to excite the fluorophore. Then, the LED was switched off and the SPAD was activated to measure the light emitted by the fluorophore. The SPAD sensor was activated 3 ns after the LED was turned off. Figure 6 shows the intensity emitted by the QD605 after exciting the 32 × 32 LEDs one by one. QD605 was detected in the two microwells as it is shown in Figure 6, such that there are five LEDs under every microwell. As can be observed, the only place where QD605 is detected is in the orange areas, corresponding to two crosses formed by the LEDs, where the microwells contain samples. The number of counts measured in the areas where QD605 was deposited is in the range from 950 to 1050. On the other areas, the number of counts measured is lower than 150. So, the device can discriminate areas with QD605 at concentration of 1 µM at low volumes (5 nL).

**Figure 7:**
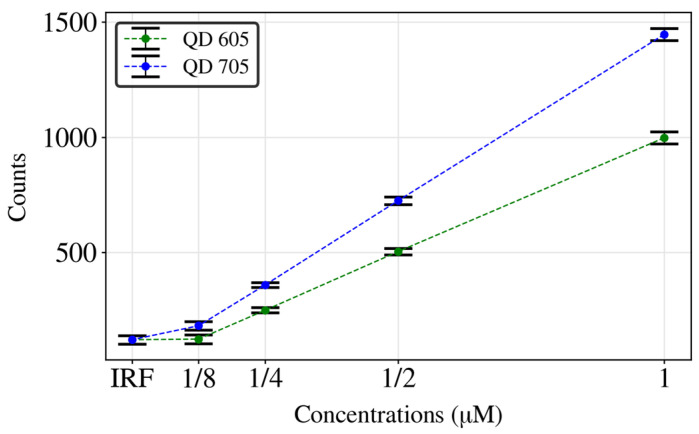
Intensity obtained in different measurements for different concentrations of QD605 and QD705.

Moreover, to test the capabilities of the device, different concentrations were measured for both QD605 and QD705, with the device being able to achieve a LoD of 1/4 µM. As can be observed, at 1/8 µM, the same number of counts are detected as in the Instrument Response (IR), i.e., the background counts when there is no fluorophore (Figure 7).

### 3.2. Time-Correlated Fluorescence Measurements

Time-Correlated Single Photon Counting (TCSPC) was used to acquire temporal information. This method consists of exciting the sample with a pulsed light source. After each excitation pulse, one of the photons emitted by the fluorophore can be detected by the SPAD sensor, which stays inhibited after the detection. The arrival time of the photon is then measured and catalogued in the corresponding histogram bin. By repeating this method several times, a histogram that represents the decay curve of the fluorophore can be reconstructed. The number of times the measurement was performed is one million, with an exposure time of each measurement of 200 ns, which makes a total exposure time of 200 ms. In this case, the number of photons detected never exceeds 5% of the total number of measurements performed (1 million measurements, maximum of 50,000 counts) in order to avoid pile-up effects [65]. In this work, the maximum counts detected for 1 µM concentration is 15,000 counts, so it can be assured that pile-up effects would not affect the device.

**Figure 8:**
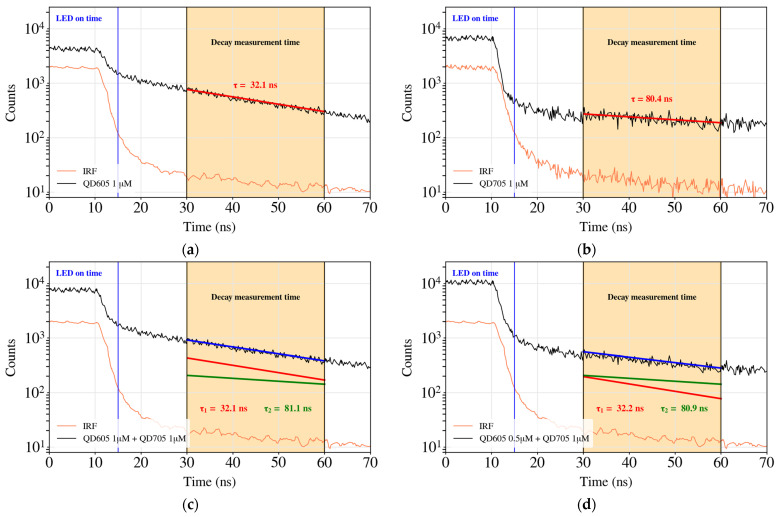
Decay time for QD605 at a concentration of 1 µM (**a**) (with A = 1953 and τ = 32.1 ns) and for QD705 also at a concentration of 1 µM (**b**) (with A = 306 and τ = 80.4 ns). In both cases, it is shown the fitted line (in red) where the amplitudes (A) and the lifetimes (τ) are obtained. (**c**,**d**) correspond to a mixture of QD605/QD705 in ratio 1 µM/1 µM and 0.5 µM/1 µM, respectively. Bi-exponential fitting results in A_1_ = 1915 and τ_1_ = 32.1 ns; A_2_ = 417 and τ_2_ = 81.1 ns in (**c**) and A_1_ = 989 and τ_1_ = 32.2 ns; A_2_ = 397 and τ_2_ = 80.9 ns in (**d**).

Figure 8 shows different reconstructed histograms corresponding to the two different fluorophores and a measurement of the Instrument Reference Function (IRF). As can be observed, the first 11 ns correspond to the LED source lighting the sample. After that, the LED is turned off and the sum of the fluorophore light and the LED light decays are detected. Then, after 3 ns, the LED is completely off (response < 3 counts). So, the influence of the LED decay is negligible and the QD fluorescence light decay can be measured. The decay of the fluorescence of QDs is described as a multi-exponential curve [66]. However, sometimes it can be approximated as a mono-exponential decay $\left(I_{fluor}=Ae^{\left(-t\backslash \tau \right)}\right)$ [24]. Figure 8 presents a linear fit of the logarithmic representation of the decay curve from 30 to 60 ns. Thus, from the inverse of the slope, the extracted lifetimes for QD605 and QD705 are of ~31.3 ns ± 0.6 ns and ~81.7 ns ± 0.9 ns, respectively, which are in good agreement with the reported values (32.7 ± 0.2 ns and 80.0 ± 3 ns, respectively) [24,63]. We also performed the analysis with a mixture of both QDs, as both fluorophores are excited by the same wavelength. Figure 8c,d shows the fit with a bi-exponential model $\left(I_{fluor}=A_{1}e^{\left(-t\backslash \tau_{1}\right)}+A_{2}e^{\left(-t\backslash \tau_{2}\right)}\right)$. Two different fluorescence lifetime channels can be selected, and we can estimate the QD605/QD705 ratio on the sample from the amplitude coefficients of both exponentials. Simple linear unmixing of the dyes can be conducted while assuming that there is no modification of the individual lifetimes [67].

**Figure 9:**
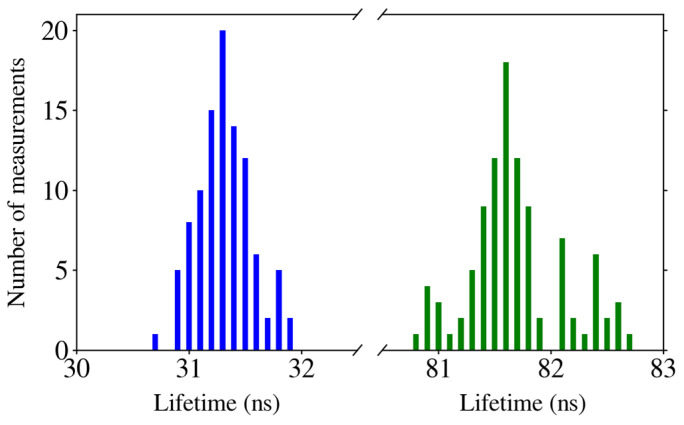
Lifetimes of QD605 (blue) and QD705 (green) for 1 µM for 100 sampled measurements to extract statistical values.

The LoD of this device using time-correlated fluorescence measurements is the same as that obtained with intensity measurements: 1/4 µM, as expected. Using the measurement method mentioned above, 100 histograms were obtained. From these histograms, the lifetime of each fluorophore was calculated for statistics. They are shown in Figure 9, where QD605 has a mean lifetime of 31.3 ns ± 0.6 ns for concentrations from 1 µM to 1/4 µM and QD705 has a mean lifetime of 81.7 ns ± 0.9 ns for concentrations from 1 µM to 1/4 µM.

**Figure 10:**
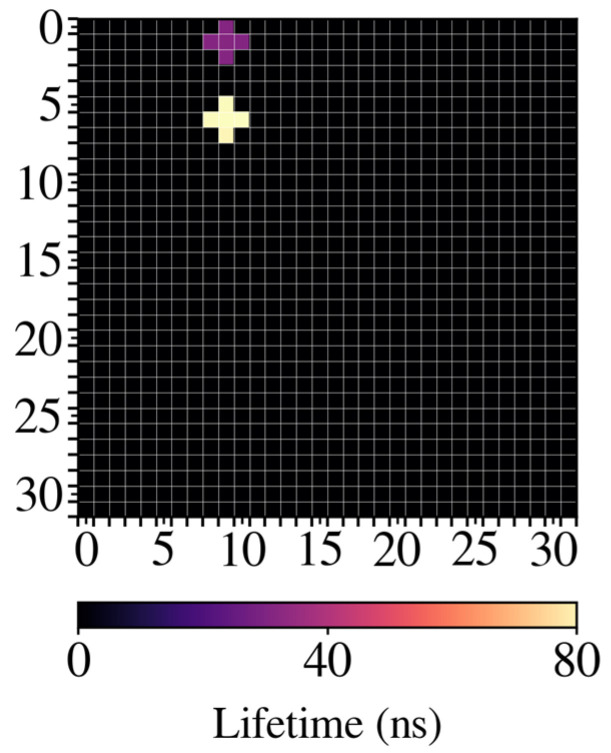
Image obtained with the device where the two fluorophores were deposited in microwells.

Figure 10 shows a representation of the lifetimes measured on top of each LED. As is clear from Figure 8, the slopes of QD705 and the IRF are very similar. To ensure that we could discern the signal from the background, bins were integrated for every LED in the range [30 ns, 60 ns], and a threshold of 5000 counts was established. Then, lifetimes were evaluated, as described previously, for curves with higher counts. In Figure 10, the zones where the fluorophores were detected correspond to the LEDs marked for QD605 and QD705. Pseudocolor was used to identify lifetimes, so that purple corresponds to QD605 and yellow to QD705. Considering the differences observed among the different fluorophores tested, we validated the instrument to develop a PoC based on fluorescence lifetime measurements using microLED arrays operating by matrix addressing.

## 4. Discussion

This research proposes a compact multiplex fluorescence detection system using an array of matrix addressable microLED arrays. The advancements in GaN technology over the past few years indicate that these devices could potentially replace lasers and other illumination sources in the field of fluorescence, particularly in PoC technology [68,69]. GaN-based devices offer simplicity, greater integration capabilities, and cost-effectiveness, making them promising alternatives. The performance of the device was validated through experiments using reference fluorophores. Furthermore, the system was employed to detect two different QD using time-correlated fluorescence measurements in the same assay. Further studies on the proposed PoC device with fluorophores bonded to antibodies and target infections are required to determine applicability. Moreover, the LoD of this setup, with its current characteristics, is 1/4 µM. Nevertheless, this does not invalidate the potential of the technique for detecting even lower concentrations, since the sensitivity depends directly on factors such as the distance between the SPAD and the sample. In this setup, the sample is located at 8 mm of the SPAD sensor, hence the low sensitivity. Some improvement could be obtained by reducing the distance between the sample and the sensors. Additionally, microlenses can be incorporated into the LED array chip to increase the optical power on the fluorophores [70]. Similarly, microlenses can be added to the SPAD array to gather the light being emitted from the fluorophores [71]. The upper LoD of this setup could also be increased from 1 µM until the pile-up effect appears (5% counts over total number of measurements) [65]. Nevertheless, to avoid pileup distortion in case it occurs at higher concentrations, we can decrease the intensity of the excitation light by controlling the bias current of the LEDs [72].

Arrays of microLEDs were used for the first time in [73]. Thus, the use of microLEDs for fluorescence detection has been implemented over and over in the past decades, but typically this has been performed with directly addressable LED arrays, which are limited by their own structure. The LED downscale size, pitch, and density in directly addressable arrays is limited by the size that the connection lines can achieve. Thus, to have drivers capable of providing the necessary speed to perform time-correlated fluorescence measurements, an array with a certain pixel and pitch size is required, or, alternatively, a low LED density on the chip. D. Bezshlyakh et al. [55] reported an array with 400 nm LED size and 400 nm distance between adjacent LEDs with a maximum number of pixels achieved of 6 × 6 due to limitations in space to connect the center pixels to the exterior. Thus, a tradeoff must be made between the size and pitch of the pixels and the size of the array. In [74], a custom chip was used to drive an array of 8 × 8 microLEDs for time-resolved fluorescence measurements. There, the microLED array was bonded by flip-chip onto the custom CMOS driver chip to obtain a direct addressing mode. The state of the art in microLED arrays driven by CMOS circuits is hybrid interconnected arrays [75]. In these devices, the CMOS driver must fit under the pixel; therefore, the smaller the pixel size and pitch is, the smaller the driver is, which limits the switching speed of the circuit. The circuits that achieved the higher speed in the field are reported by J. Canals et al. [76] and N. B. Hassan et al. [77], achieving maximum speeds of 1 MHz, which makes these devices unsuitable for time-resolved fluorescence measurements. Thus, this approach is a tradeoff between driving capabilities, due to the size of the driving circuit that is below the LED, and the density and size of LEDs. Given that the trend of microLED technology for displays is to make smaller microLEDs integrated in higher density arrays, it is a limitation for the use of this addressing mode. On the other hand, there is no limit on the size of the driving circuitry for matrix addressable driving since there is a driver for each column and row of the array that does not need to be under the LED pixel. This allows the driver to provide enough driving current for fluorescence excitation and to be as large as necessary to achieve the driving speeds required for time-resolved fluorescence.

Table 1 summarizes microLED arrays for framerate and array size used for different fields. It can be observed in the tradeoff between array and pixel size, power, and speed. An array with high PPI used for display applications is presented [78] for comparison. Such an array has 1920 × 1080 pixels, but with a limited speed of 125 fps. On the high current side, Poher et al. [79] described a matrix addressable array of 64 × 64 used for neuron stimulation. They achieve high optical power by driving the LEDs up to 10 mA, but with a limited speed of 600 fps.

In this work, we propose the use of microLEDs for multiplexed time-resolved fluorescence in PoC devices. It is centered on matrix addressable arrays that allow for high integration, with the only limit being on the pixel pitch and size, which is determined by the GaN technological limit. Moreover, the driving circuit can be placed outside the microLED array, which eliminates the limitation of the driver size that appears in hybrid interconnected arrays, thus allowing us to design the driver to achieve the rates required for time-resolved fluorescence measurements.

In addition, the research presented in this work paves the way for the development of miniaturized microscopes [80,81,82,83,84] based on fluorescence, the gold standard tool used in biology. This promising advancement is envisaged through the utilization of large arrays integrating smaller LEDs, complemented by the appropriate driving circuits.

## 5. Conclusions

In this paper, we present a fluorescence detection device that allows for both intensity and lifetime measurements. The device is small and easy to assemble, achieved by joining the advantages of a camera with high SNR CMOS SPAD detectors with an array of microLEDs, which provide high optical power and fast switching speed. Moreover, by using time-gating with the SPAD, the device avoids the use of any optical filter to isolate the fluorescence intensity from the LED light. This is possible thanks to the measurement being taken after the LED is turned off. However, with the auxiliary electronics used to control the device, this approach has some limitations. For lifetime evaluation, it restricts the use of fluorophores to those with decay times longer than ~10 ns. This limits the use of the PoC for organic fluorophores, with lifetimes well below 5 ns. To address such range, new arrays with smaller LED size can be developed to reduce the parasitic capacitance and decrease the switching response. Additionally, more efficient LED drivers with improved switching times can be produced. Nevertheless, addressing such lifetimes could be difficult for a miniaturized microLED-based PoC.

The results obtained by the system endorse that it can detect fluorophores in intensity mode at a high speed, and, moreover, it can detect different fluorophores in the same measurement by using the time-resolved fluorescence method. All of this can be achieved while operating with very small samples volumes (5 nL). This device holds high potential for applications in the scan of biological samples, analytical laboratories, and for clinical diagnosis.

Furthermore, the device enables the possibility of advances in different fields, such as building a fluorescence microscope by using an array of microLEDs [85] or building multi-well detection devices for multiplexed assays.

## Figures and Tables

**Table 1:** Comparison of GaN microLED arrays driven by CMOS circuits.

Reference	[77]	[76]	[78]	[79]	[74]	This Work
Driver type	in-pixel	in-pixel	in-pixel	MA	DA	MA
Application	display	display	display	neuron stimulation	fluorescence	fluorescence
Resolution	128 × 128	512 × 512	1920 × 1080	64 × 64	8 × 8	32 × 32
Pixel pitch	50 µm	18 µm	2.5 µm	40 µm	200 µm	100 µm
Pixel density	508 PPI	1411 PPI	10,000 PPI	635 PPI	127 PPI	254 PPI
Switch speed	83 kfps	1 MHz–9.15 kfps	n.a.	600 fps	1.28 GHz	500 MHz
Max. LED current	87 µA	120 µA	1.6 µA	10 mA	n. a.	20 mA
LED bias voltage	5 V	up to 5V	n.a. V	up to 4V	up to 5V	up to 10 V
CMOS Tech. Node	0.18 µm	0.18 µm	n.a.	n.a.	0.35 µm	0.35 µm

## Data Availability

The original contributions presented in the study are included in the article, further inquiries can be directed to the corresponding authors.
